# How incidental and intentional news exposure in social media relate to political knowledge and voting intentions

**DOI:** 10.3389/fpsyg.2023.1250051

**Published:** 2023-12-21

**Authors:** Jana H. Dreston, German Neubaum

**Affiliations:** Department of Human-Centered Computing and Cognitive Science, University of Duisburg-Essen, Duisburg, Germany

**Keywords:** subjective knowledge, political knowledge, social media, election, incidental news exposure

## Abstract

**Background:**

Citizens are expected to make informed voting decisions. Theoretical approaches suggest that people are most likely to acquire their political knowledge through media. As more people turn to social media as a source of news, the political knowledge gains from using these technologies are called into question. Previous research has shown that rather than increasing objective political knowledge, the use of social media for news only increases people’s metacognitive sense of being knowledgeable (subjective knowledge), which in turn increases their political participation. However, it remains to be understood which particular forms of social media use, e.g., incidental or intentional news exposure, are related to which dimension of political knowledge. The present work examines (a) the extent to which different motivational forms of social media news consumption foster subjective knowledge, and (b) whether this metacognition is related not only to political participation as a broad concept, but also to specific democratic outcomes such as voting intentions.

**Methods and results:**

Results from a pre-registered, pre-election survey (*N* = 1,223) of social media users show that intentional news seeking, but not incidental news exposure on social media, is directly related to increased subjective knowledge. Subjective knowledge appears to explain the relationship between social media news use and voting.

**Discussion:**

By showing that incidental and intentional social media news use affect subjective knowledge differently, this study provides preliminary and nuanced insights into the ultimate role that social media technologies can play in democratic processes.

## 1 Introduction

Political participation, such as voting, is at the very heart of democracy (Makarenko, 2015). According to democratic ideals, citizens are expected to make informed voting decisions based on the reflective handling of political knowledge. Presumably, the media are providers of this knowledge (Norris, 2000). However, corresponding research such as the O-S-R-O-R model (Cho et al., 2009) suggests that it is not media-induced political knowledge but people’s reasoning (the processing of new information and forming of associations with existing knowledge) about news that is important for political participation.

Social media technologies, which have become established sources of political news, enable different forms of reasoning, either in the form of political talk with others (“interpersonal reasoning”) or in relation to oneself and one’s perspective on the topic (“intrapersonal reasoning”). While corresponding research has considered social media news use as a very broad operationalization of media use (Boulianne, 2015; Lee et al., 2022), not all modes of news acquisition offer equal opportunities for cognitive elaboration, a form of reasoning. The relationship between intentional news search and cognitive elaboration has long been established (Eveland, 2001). In addition, recent research shows that incidental news exposure on social media can also lead to cognitive elaboration (Oeldorf-Hirsch, 2018). However, it is unclear which forms of social media use lead to which forms of reasoning and participation.

To this end, the present work aims to extend the state of knowledge on two levels: First, we focus on the question of *when* social media can lead to political participation. Taking into account the different motivational states of news exposure on social media (Oeldorf-Hirsch, 2018; Matthes et al., 2020), we distinguish between intentional versus incidental news exposure in social media. We investigate which forms of news exposure are associated with knowledge gains and reasoning that could influence subsequent political participation in the form of voting intentions.

Second, we turn to the question of *how* social media relate to political participation. While social media allow for interpersonal and intrapersonal reasoning, the O-S-R-O-R model suggests that only interpersonal reasoning in the form of political messaging and political talk positively affects knowledge gains and participation (Cho et al., 2009). However recent research, suggests that the effect of social media on political participation can be explained by subjective knowledge (Raju et al., 1995; Yamamoto et al., 2018; Lee et al., 2022). The latter has been conceptualized as the self-assessed ability to recall and judge one’s own knowledge (Radecki and Jaccard, 1995; Frauhammer and Neubaum, 2023) and may be viewed as an outcome of intrapersonal reasoning. Focusing on how different forms of reasoning – enabled by social media use – affect subsequent voting intentions will provide further insights into the complex relationships between social media and political participation.

Building on the above, the present work applies the established O-S-R-O-R model (Cho et al., 2009) to the social media context and extends it by disentangling how different forms of media use can lead to reasoning and knowledge acquisition, thereby fostering political participation.

## 2 Literature review

### 2.1 The relationship between online media, knowledge, and participation

When it comes to explaining people’s political engagement in relation to their media use, the O-S-R-O-R model (Cho et al., 2009) provides preliminary predictions about the psychological antecedents of political participation. Here, the first “O” refers to “Orientations,” structural, cultural, cognitive, and motivational characteristics of the audience (Markus and Zajonc, 1985). These influence the way people consume news in the media, which serves as the “Stimulus.” This leads to “Reasoning,” which describes interpersonal and intrapersonal processes of cognitive elaboration (Shah et al., 2007). These lead to “Outcome Orientations” such as objective knowledge and “Responses” such as political participation. Broadly consistent with these predictions, numerous studies have shown that social media news consumption is related to political participation (see Boulianne (2015) for a meta-analysis). However, there is no direct relationship from one to the other, but the relationship is mediated through different forms of reasoning. The “black box” between social media use and participation has yet to be uncovered.

Social media platforms differ in many ways from the online news analyzed in the original O-S-R-O-R model (Cho et al., 2009), potentially challenging some of the model’s predictions and implications for how political knowledge can be gained through social media (Shah et al., 2017). While news in traditional online news outlets is largely produced by trained journalists, social media allows anyone to contribute to the endless flow of news. People can share news, produce their own news, and even express their opinions about the news as a form of political talk. While users can access full articles in traditional online media, posts on social media posts often contain scarce, superficial information (Schäfer, 2020).

The O-S-R-O-R model (Cho et al., 2009) emphasizes the process of reasoning as crucial in explaining the impact of online news media on objective knowledge and political participation. It distinguishes between cognitive elaboration and collective consideration (Shah et al., 2007). Cognitive elaboration is an intrapersonal process, in which people reflect on news content and integrate it with existing knowledge (Eveland, 2001). Political discussion and messaging, on the other hand, are forms of collective consideration that involve both interpersonal and intrapersonal modes of thinking (Cho et al., 2009).

An understudied aspect of the O-S-R-O-R model (Cho et al., 2009) relates to the first “O,” the orientations that news consumers bring to the table when consuming news. The model operationalizes this as the likelihood of encountering political advertisements. In doing so, it largely underspecifies people’s motivations for encountering political news. In line with research on incidental news exposure in social media (Knoll et al., 2020; Matthes et al., 2020; Shahin et al., 2021), we argue that motivation, as manifested in the intention to seek news or not, is critical for (a) news processing and (b) consequential effects such as knowledge and participation. In the following, we first consider the extent to which social media use promotes political knowledge and political talk, and then how these associations might be attributed to *how* people use social media.

### 2.2 (Subjective) political knowledge

Political knowledge can be studied along at least two dimensions: *objective* knowledge and *subjective* knowledge. We argue that while both forms of knowledge are related, they represent unique dimensions. Objective knowledge describes a potential outcome of news consumption, whereas subjective knowledge describes the perception of one’s own knowledge.

With the rise of social media, studies have examined whether receiving news from social media can lead to objective knowledge gains as it does when consuming traditional media (Eveland, 2001; Sotirovic and McLeod, 2001; Cho et al., 2009). While results have been mixed (Van Aelst et al., 2022), a recent meta-study found no effect of global social media use on objective political knowledge (Amsalem and Zoizner, 2022). Several reasons for the lack of effect have been discussed. Selective exposure and echo chambers in social media may reduce people’s opportunity to detect and internalize new information (Cargnino and Neubaum, 2021). In addition, the combination of different news platforms may lead to information overload, which in turn may lead to news avoidance on social media (Park, 2019; Granderath et al., 2021). Most information on social media is reported superficially as so-called snack news, which conveys little actual information (Schäfer, 2020; Ohme and Mothes, 2023). This, in turn, may lead to little elaboration and therefore little knowledge gain (Petty and Cacioppo, 1986). In addition, social media allows for the rapid spread of misinformation (van Erkel and Van Aelst, 2021; Van Aelst et al., 2022), which may naturally limit the potential growth of accurate political knowledge.

Among different disciplines, the metacognition of one’s own knowledge is known as subjective knowledge (Carlson et al., 2009; Yamamoto et al., 2018), perceived knowledge (Schäfer, 2020), and confidence in knowledge (Lee and Matsuo, 2018). We will refer to it as subjective knowledge. As a perception, it is influenced not only by one’s objective knowledge (Carlson et al., 2009), but also by characteristics of the learning environment or the news stimulus itself (Ran et al., 2016; Weber and Koehler, 2017).

The O-S-R-O-R model (Cho et al., 2009) defines intrapersonal reasoning as the integration, reflection, and sensemaking of media content within one’s cognitive system. We believe that subjective knowledge is a direct result of the intrapersonal reasoning process as subjective knowledge describes the perceived ability to access and express information stored in one’s memory (Radecki and Jaccard, 1995). When people encounter news on social media, they engage in intrapersonal reasoning by comparing the incoming information with the information stored in their memory. Because most news posts on social media convey little information (Schäfer, 2020), they do not have the capacity to increase people’s objective knowledge, but they still lead to intrapersonal reasoning, which can be observed in an increased (subjective) sense of being knowledgeable (Yamamoto et al., 2018; Lee et al., 2022).

The architecture of social media platforms supports repeated exposure to similar news. Repeated posts on the same topic, even if they do not provide additional information, can create familiarity that people heuristically mistake for objective knowledge, when in fact only one’s subjective knowledge has increased (Metcalfe et al., 1993; Müller et al., 2016; Schäfer, 2020). This discrepancy between actual and perceived learning can create a so-called illusion of knowledge (Schäfer, 2020; Lee et al., 2021). Based on these theoretical lines and empirical findings, we hypothesize:

*Hypothesis 1* (*H1*): The frequency of social media news use is positively associated with subjective political knowledge.

### 2.3 Political talk

Numerous lines of research have documented the role of political talk for learning in online and offline settings (Eveland et al., 2004; Cho et al., 2009). Both interpersonal and intrapersonal aspects of cognitive elaboration play a key role (Cho et al., 2009). Both anticipating political talk and actually discussing with others lead to deeper processing, which in turn facilitates learning (Eveland et al., 2004). In particular, confirmatory feedback and cueing are critical (Moore and Coronel, 2022). In offline settings, neither network size nor network heterogeneity mattered for learning, but only the mere frequency of political talk (Amsalem and Nir, 2019). Social media, however, expands the opportunities for political talk. Users can engage in asynchronous discussions with more than one partner, express their views by sharing content, or comment on a post. As observed in offline discussions, online political talk leads to elaboration (Oeldorf-Hirsch, 2018; Hopp et al., 2023).

Talking about politics may not only affect one’s discussion partner, but also oneself. This type of effect has been termed as self-effect (Valkenburg, 2017). Accordingly, exchanging with others about politics may not only promote the elaboration of the topic, but also change one’s self-concept. If one participates in a political discussion online, it’s very likely that one will see oneself as a politically knowledgeable person, because it is obvious that discussions should be based on knowledge. This effect is amplified in public, as public actions are more accessible to memory scans, which in turn inform one’s self-concept (Bem, 1972; Valkenburg, 2017; Ward et al., 2022). While these self-directed effects of media-induced political talk were recognized by the authors of the O-S-R-O-R model (Cho et al., 2009), they were never explicitly hypothesized in the model itself. Therefore, we argue that subjective knowledge as a direct result of intrapersonal reasoning is a separate step in this process.

Furthermore, it can be assumed that political talk leads to familiarity with the topic being discussed (Choi et al., 2017). Both familiarity and repetition serve as heuristics for assessing one’s subjective knowledge (Metcalfe et al., 1993; Müller et al., 2016; Schäfer, 2020). While the separate links between political talk and both objective and subjective knowledge have not yet been empirically tested, we draw on the notion outlined above and propose:

*Hypothesis 2* (*H2*): Political talk on social media is positively associated with (a) objective political knowledge and (b) subjective political knowledge.

### 2.4 Intentional news search and incidental news exposure

Extending the predictions of the O-S-R-O-R model (Cho et al., 2009), the present work argues that *the motivation with which people use social media* platforms makes a difference for their (perceived) knowledge gains. To analyze the effects of social media news use, studies often measure the total frequency of use (Müller et al., 2016; Strauß et al., 2021). However, this approach largely neglects *how* people use these platforms and *what* they see on them (Knoll et al., 2020). Therefore, we distinguish between intentional news search and incidental news exposure as two forms of motivational orientations.

Motivation has long been recognized as crucial to learning from online news because it can stimulate elaboration, which in turn leads to objective learning (Luskin, 1990; Delli Carpini and Keeter, 1996; Eveland, 2001). Social media create the possibility of many unsolicited, incidental encounters with news. Not all of these lead to the same knowledge gains. Shahin et al. (2021) found that intentional search led to more elaboration than incidental exposure because it triggered a peripheral route of elaboration compared to the central route triggered by intentional search. The political incidental news exposure model (Matthes et al., 2020) suggests that people engage in relevance appraisal for any post they encounter incidentally on social media that does not match their initial processing goals (e.g., entertainment). For stimuli judged to be relevant, resources are allocated, and elaboration can occur. However, few cognitive resources are allocated to stimuli that are perceived as irrelevant (Matthes et al., 2020). Due to the lack of elaboration, learning effects for non-relevant stimuli would be rather small.

Results on the relationship between incidental news exposure and objective knowledge are mixed. While Weeks et al. (2021) showed that incidental news exposure is particularly beneficial for people with otherwise low motivation to search for news, other studies found no learning effect (Bode, 2016; Shehata and Strömbäck, 2021). In line with the O-S-R-O-R model (Cho et al., 2009), Oeldorf-Hirsch (2018) found that incidental news exposure only leads to elaboration through political talk, but not directly by itself. However, in contrast to broad social media news, this elaboration is not sufficient to lead to objective knowledge. This may explain the lack of effects in some studies that did not specify *how* individuals use these media. In contrast, other studies have found that even low levels of elaboration induced by incidental exposure are associated with objective learning (Nanz and Matthes, 2022b). In addition, a meta-analysis by Nanz and Matthes (2022a) found a positive association between incidental news exposure and objective knowledge. However, they did not take depth of elaboration induced by relevance appraisal into consideration. When it comes to how incidental news exposure affects subjective political knowledge, empirical evidence is scarce: An initial study suggests that superficial processing is negatively associated with subjective knowledge, while deeper elaboration is positively associated with subjective knowledge (Nanz and Matthes, 2022b).

It is well documented that intentional news seeking in traditional media leads to learning through attention and elaboration (Eveland, 2001). However, when it comes to the use of social media, studies suggest that while intentional news searching may lead to attention and elaboration, it still seems to be unrelated to objective knowledge (Oeldorf-Hirsch, 2018). Following the idea of altered self-concepts (Valkenburg, 2017), people may see themselves as more knowledgeable by observing their news-seeking behavior. This may be true even in situations where one does not gain any objective knowledge. Because the motivational orientation differs between incidental news exposure and intentional news search, we ask:

Research question 1 (RQ1): Are incidental news exposure and intentional news exposure on social media differently associated with subjective political knowledge?

### 2.5 Social media and political participation

According to democratic ideals, the ultimate purpose of acquiring political knowledge is to participate in political processes as an informed citizen. Political participation is defined as voluntary, nonprofessional actions intended to influence politics (Verba and Nie, 1972; Brady, 1999). Democratic voting represents a classic example of institutional and conventional political participation (van Deth, 2016).

When do people decide to vote? To date, many models conceptualize different influences on individual voter turnout. Some view voting as a social behavior. People vote because they are asked to do so or because they perceive voting as a social norm (Gerber and Green, 2000; Arceneaux and Nickerson, 2009; Gerber and Rogers, 2009). Traditional media is a driving force for both (Smets and van Ham, 2013). Many studies have linked the use of social media to broad political participation (Boulianne, 2009, 2020). While most political participation is costly, requiring resources such as time and money (Verba et al., 1995), social media reduce the cost of many forms of political participation. Liking and commenting require minimal effort. In contrast, the act of voting (or even registering to vote in some countries) requires resources such as time, transportation, or childcare (van Deth, 2016; Knoll et al., 2020). People with more resources are therefore more likely to participate in elections (Verba and Nie, 1972). While the potential mobilizing nature of social media for voter turnout is documented (Haenschen, 2016), the real-life relationship between the two remains unclear (Zhuravskaya et al., 2020).

According to the O-S-R-O-R model, political talk is essential in explaining media effects on participation (Cho et al., 2009). Talking to politically active people increases the likelihood of learning about political activities in which one might like to participate (Verba et al., 1995; Xenos and Moy, 2007). It can also convey participatory norms that increase voter turnout (Gerber and Rogers, 2009). However, research on the effect of political talk on voter turnout is inconclusive (Smets and van Ham, 2013). Based on the argument that political talk may provide people with greater (subjective) knowledge and interest in politics, we expect political talk to be positively associated with voting intentions.

*Hypothesis 3* (*H3*): Both (a) social media news use and (b) political talk are positively correlated with voting intentions.

### 2.6 Explanations for the social media–voting link

In an integrative model, Lee et al. (2022) describe the mediating effect of social media induced subjective knowledge on political participation. Further supporting previous evidence, this model finds no effect of social media news use on objective knowledge, nor any effect of objective knowledge on political participation (Sotirovic and McLeod, 2001; Cho et al., 2009; Amsalem and Zoizner, 2022). However, using social media to get news increased subjective knowledge. In turn, the perception of being informed increased the likelihood of political participation.

In contrast to these findings on political participation, theories of voting behavior describe objective knowledge as both a resource and an antecedent of voting (Smets and van Ham, 2013). These contrasting conceptions of the role of objective knowledge suggest differences between broad political participation and voting. Furthermore, objective and subjective knowledge may play different roles in the process of voting.

First, objective political knowledge seems to be a prerequisite for voting in line with one’s personal interests (Smets and van Ham, 2013; Lee and Matsuo, 2018). Second, subjective knowledge is a stronger predictor of people’s final actions than objective knowledge. Lack of perceived knowledge is one of the main reasons for young people to obtain from voting and explains as much as 10% variance in their voter turn-out (Kaid et al., 2000, 2007). Because subjective knowledge is more assessable in most behavioral situations (Dunning, 2011), people will rely on their subjective rather than objective knowledge when assessing whether they feel knowledgeable enough to vote or participate politically (Almond and Verba, 1965; Weber and Koehler, 2017; Schäfer, 2020).

*Hypothesis 4* (*H4*): Subjective political knowledge is positively associated with voting intentions.

*Hypothesis 5* (*H5*): Subjective political knowledge mediates the positive relationship between (a) social media news use and (b) political talk and voting intentions.

The social media political participation model (Knoll et al., 2020) expects that intentional news search, but not incidental news exposure, is related to high-effort political participation such as voting. However, initial research has found that incidental news exposure is also related to both online and offline political participation (Nanz and Matthes, 2022b). In contrast, Shahin et al. (2021) show that incidental news exposure is only related to online participation, while intentional news search is related to both online and offline participation. Based on the O-S-R-O-R model (Cho et al., 2009), one could argue that higher motivation to consume information in social media has a positive effect on political participation by improving reasoning. The effect may be weaker for incidental exposure as a manifestation of low motivation to consume news. Therefore, we contrast both incidental news exposure and intentional news search in their relationship with voting intentions.

Research question 2 (RQ2): Are incidental news exposure and intentional news search differently associated with voting intentions?

Research question 3 (RQ3): Does subjective political knowledge mediate the positive relationship between (a) incidental news exposure and (b) intentional news search and voting intentions?^1^^,^^2^

## 3 Methods

### 3.1 Participants and procedure

A power analysis based on Lee et al. (2022) using *pwrSEM* (Wang and Rhemtulla, 2021) and power4SEM (Jak et al., 2021) indicated that a sample size of *N* = 1,050 would be sufficient to detect direct relationships of interest smaller than 0.1 with a power of 0.9 and relationships smaller than 0.3 with a power of 0.99. *Post hoc* power analyses confirmed that the sample size was sufficient to detect all hypothesized direct effects with a power of >0.99.

We conducted a pre-registered^3^ cross-sectional online survey in May 2022, 2 weeks before the regional elections in the state of North Rhine-Westphalia (NRW), Germany. This survey was approved by the local institutional ethics review board. Participants provided written informed consent to participate in the study. The final sample (*N* = 1,223) was representative of social media users in NRW. Participants were aged 18–69 years (*M* = 41.09, *SD* = 14.94), 47.26% were female (52.41% male, <1% diverse, <1% no answer), and most were employed (66.64%). They were relatively well educated (63.21% have a higher educational degree) and 87% used social media daily. Most participants classified themselves to be politically centrist.

### 3.2 Measures

#### 3.2.1 Social media news use

On a scale of 1 (never) to 7 (always), we asked people how often they used social media to stay “informed about current events and public affairs,” “get news about current events from mainstream media,” and “get news from online news sites” (Gil de Zúñiga et al., 2017)^4^; Cronbach’s α = 0.91; McDonald’s Ω = 0.87; *M* = 4.14, *SD* = 1.62.

#### 3.2.2 Incidental news exposure

We measured incidental news exposure by asking participants how often they “come across political news in your social media news feeds (without actively searching for it)” (Tewksbury et al., 2001; Oeldorf-Hirsch, 2018) on a scale of 1 (never) to 7 (always); *M* = 4.02, *SD* = 1.53.

#### 3.2.3 Intentional news search

Intentional news search was measured by asking how often they “actively seek out political news on social media channels” on a 7-point Likert scale ranging from 1 (never) to 7 (always); *M* = 3.4, *SD* = 1.74.

#### 3.2.4 Political talk

We measured political talk by asking how often participants “discuss political news on social media,” “forward political news on social media,” and “comment on political news on social media” on a scale from 1 (never) to 7 (always); Cronbach’s α = 0.91; McDonald’s Ω = 0.91; *M* = 2.49, *SD* = 1.5.

#### 3.2.5 Objective political knowledge

The operationalization of objective knowledge varies widely across studies (Barabas et al., 2014). By definition, no scale can capture the full construct of political knowledge. However, Delli Carpini and Keeter (1996) have shown that a small number of political knowledge questions can capture much of the variance in political knowledge. In doing so, these questions function as indicators of knowledge beyond the questions asked. However, most knowledge questions can be organized along two axes, a temporal and a topical axis. On the temporal axis, knowledge is classified as either recent (surveillance) or past (static). Along the topical axis, knowledge is classified as either focused on individuals and institutions (general) or policy (Barabas et al., 2014). One example question to measure surveillance general knowledge was “What is the name of the SPD’s front-runner for the state election?,” while a static policy question was “In which year did Angela Merkel announce the ending of nuclear power stations in Germany?.” To cover the full range of objective political knowledge, items from all four categories were pretested (see Supplementary Table S5). Single-choice questions focused on either Germany or NRW. Correct answers were coded as 1 and incorrect answers were coded as 0.

A pretested set of four questions from each category contained two questions about Germany and two questions about NRW. A confirmatory factor analysis reduced the number of items to 12 that loaded on one factor. The model fit was good (CFI = 0.98; RMSEA = 0.02; Guttman’s lambda = 0.78; *M* = 0.56, *SD* = 0.24).

#### 3.2.6 Subjective political knowledge

To align with the four objective political knowledge categories (Barabas et al., 2014), subjective political knowledge was measured analogously. Participants were asked how much they knew about each knowledge category on a 5-point scale, ranging from 1 (not at all) to 5 (completely). Each dimension was measured with a focus on Germany and NRW. Confirmatory factor analysis reduced the original eight items to five, all of which loaded on one factor. The final scale consisted of all four NRW-focused items plus one item focusing on surveillance policy in Germany. The model fit was good (CFI = 0.99; RMSEA = 0.08; Cronbach’s α = 0.9; McDonald’s Ω = 0.92).

#### 3.2.7 Voting intentions

Participants’ intentions to vote in the upcoming state election were measured on a scale of 0–100%. Participants who had already voted by absentee ballot were asked to report 100% certainty.^5^ Voting intentions were high with 65.99% reporting that they were 100% certain to vote (*M* = 84.41, *SD* = 29.63).

## 4 Results

Our hypotheses testing includes (1) a conceptual replication of Lee et al. (2022), which relies on social media news use as a broader form of media exposure (Model 1) and (2) an extension of previous research by distinguishing between intentional and incidental news exposure (Model 2). In Model 2, we controlled for age, gender, education, and political interest, but in Model 1 the introduction of control variables reduced model fit below thresholds^6^. Individual analyses for each form of social media news use can be found in the Supplementary Figures S2–S4. Both models fit the data adequately. Structural equation models were calculated using the lavaan package in R (Rosseel, 2012).

We found support for *H1* stating that social media news use was associated with increased subjective knowledge (*β* = 0.21, *p* < 0.001). *H2* was partly supported as political talk was (a) negatively associated with objective knowledge (*β* = −0.24, *p* = 0.001), but (b) positively associated with subjective knowledge (*β* = 0.14, *p* < 0.001). Addressing RQ1, while incidental news exposure was unrelated to subjective knowledge (*β* = −0.02, *p* = 0.595), intentional news search (*β* = 0.09, *p* = 0.008) was a positive predictor. A subsequent comparison of correlations showed that the correlation between intentional news search and subjective knowledge (*r* = 0.32, *p* < 0.001) was significantly higher compared to the relationship between incidental news exposure and subjective knowledge (*r* = 0.18, *p* < 0.001); *z* = 6.41, *p* < 0.001.^7^ Neither incidental news exposure (*β* = 0.05, *p* = 0.186), nor intentional news search were directly related to objective knowledge (*β* = −0.01, *p* = 0.805).

**Figure 1:**
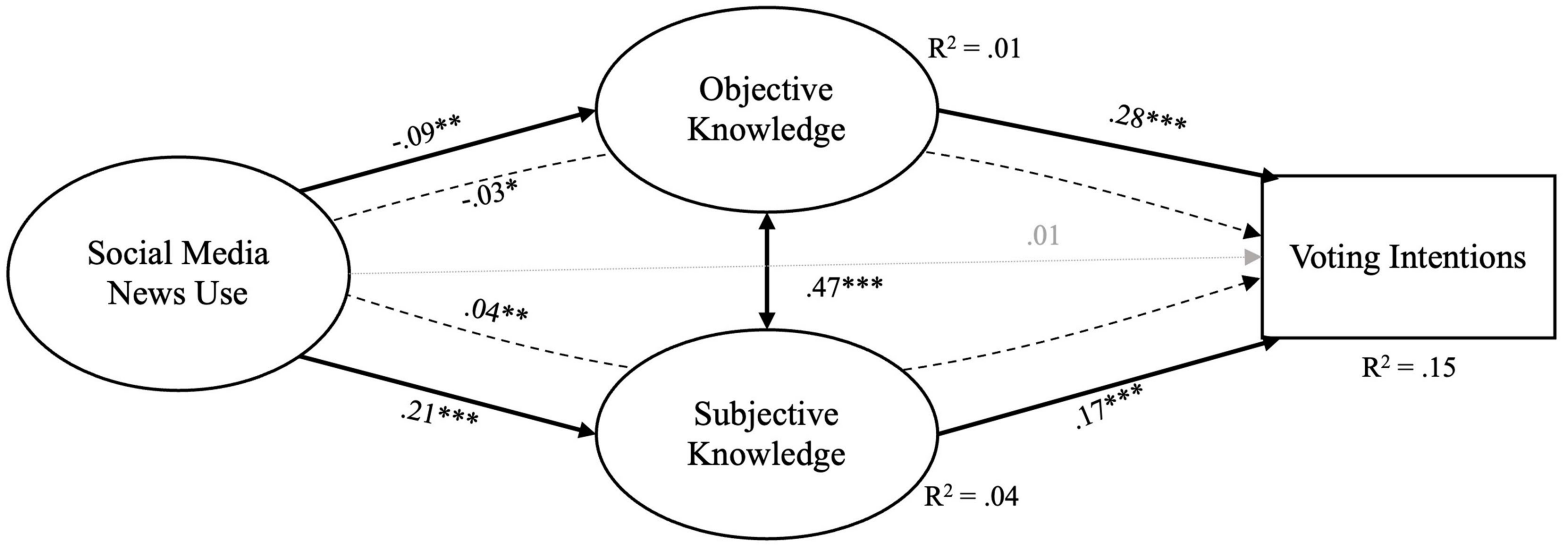
Model 1. Model fit: *χ*^2^(270) = 366.55, *p* < 0.001, *χ*^2^/df = 1.75, CFI = 0.98, RMSEA = 0.03 (90% CI: 0.02, 0.03), SRMR = 0.03; *p* < 0.05 (*), *p* < 0.01 (**), *p* < 0.001 (***); Full lines indicate significant direct associations, dashed lines indicate indirect significant associations, dotted lines indicate non-significant association.

**Figure 2:**
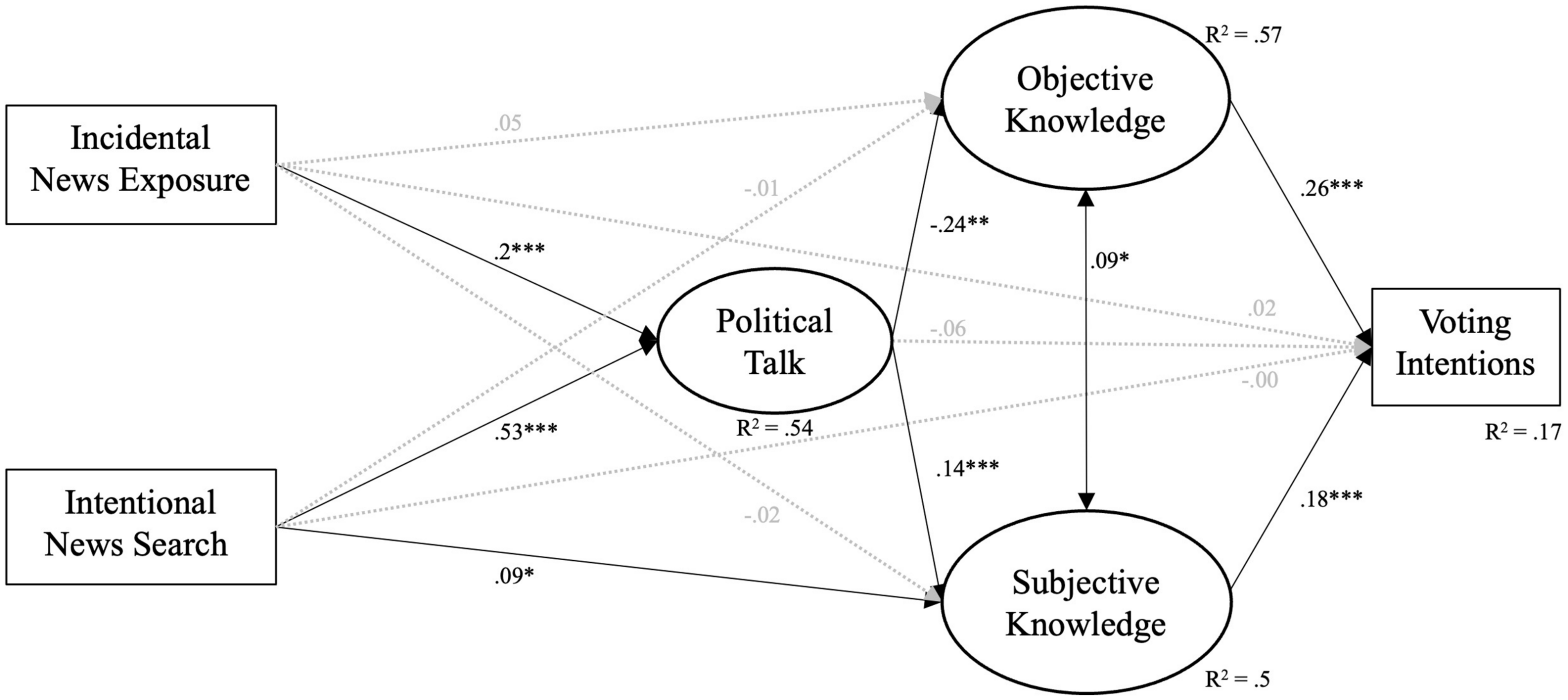
Model 2. Model Fit: c2(286) = 912.761 *p* < 0.001, c2/df = 3.191, CFI = 0.95, RMSEA = 0.04 (90% CI.: 0.039, 0.045), SRMR = 0.04; *p* < 0.05 (*), *p* < 0.01 (**), *p* < 0.001 (***); controlled for age, gender, education, and political interest. Full lines indicate significant direct associations, dotted lines indicate non-significant association.

Neither (a) social media news use (*β* = −0.01, *p* = 0.796) nor (b) political talk (*β* = −0.06, *p* = 0.157) were related to voting intentions. Therefore, we rejected *H3*. Similarly, neither incidental news exposure (*β* = 0.01, *p* = 0.677) nor intentional news search (*β* = −0.01, *p* = 0.896) were related to voting intentions (addressing RQ2). In support of *H4*, we found subjective knowledge to be associated with increased voting intentions (Model 1: *β* = 0.17, *p* < 0.001; Model 2: *β* = 0.18, *p* < 0.001). Additionally, objective knowledge was positively associated with increased voting intentions (Model 1: *β* = 0.282, *p* < 0.001; Model 2: *β* = 0.26, *p* < 0.001; Figures 1, 2).

In line with *H5*a, subjective knowledge served as a mediator for the relationship between social media news use and voting intentions (*β* = 0.03, *p* = 0.001). Additionally, subjective knowledge mediates the relationship between political talk and voting (*β* = 0.03, *p* = 0.004), supporting *H5*b. Both political talk and subjective knowledge mediate the relationship between (a) incidental news exposure and voting (*β* = 0.005, *p* = 0.008) and (b) intentional news search and voting intentions (*β* = 0.013, *p* = 0.005), answering RQ3. For estimates and 95% confidence intervals see Supplementary Table S6.

For all zero-order correlations of objective and subjective knowledge categories see Supplementary Table S1.

## 5 Discussion

The aim of this research was to investigate *when* and *how* social media use is related to political participation. Building on the O-S-R-O-R model (Cho et al., 2009), we examined the role of (a) motivation, as seen in the O-S conjunction, distinguising between incidental and intentional news exposure, and (b) extending our understanding of reasoning, by including subjective knowledge as a manifestation of intrapersonal reasoning. We show that motivation matters for the depth of reasoning, as intentional search is more strongly related to political talk than incidental exposure. Furthermore, only intentional search is directly related to subjective knowledge, whereas for incidental exposure, political talk mediates the relationship with subjective knowledge. Consistent with O-S-R-O-R (Cho et al., 2009), reasoning (as subjective knowledge, but not political talk) is related to both objective knowledge (O) and voting intentions (R). Our results support the notion that reasoning (either through political talk or intrapersonal reflections) is key for social media to be positively related to political participation (Table 1).

**Table 1:** Zero-order correlations between all measured constructs.

Variable	*M*	*SD*	Skewness	Kurtosis	1	2	3	4	5	6	7
1. Age	41.09	14.94	0.16	−1.25							
2. Social Media News Use	4.02	1.53	−0.41	−0.41	−0.24^**^ [−0.30, −0.19]						
3. Incidental News Exposure	4.02	1.53	−0.41	−0.41	−0.24^**^ [−0.30, −0.19]	0.64^**^ [0.60, 0.67]					
4. Intentional News Search	3.40	1.74	0.08	−1.02	−0.14^**^ [−0.19, −0.08]	0.72^**^ [0.70, 0.75]	0.63^**^ [0.60, 0.66]				
5. Political talk	2.49	1.50	0.81	−0.26	−0.19^**^ [−0.24, −0.14]	0.55^**^ [0.51, 0.59]	0.56^**^ [0.52, 0.59]	0.68^**^ [0.65, 0.71]			
6. Objective knowledge	0.56	0.24	−0.02	−0.95	0.36^**^ [0.31, 0.41]	−0.08^**^ [−0.13, −0.02]	−0.07^**^ [−0.13, −0.02]	−0.01 [−0.06, 0.05]	−0.09^**^ [−0.15, −0.04]		
7. Subjective knowledge	3.16	0.87	−0.49	−0.16	0.19^**^ [0.14, 0.25]	0.19^**^ [0.14, 0.24]	0.18^**^ [0.12, 0.23]	0.33^**^ [0.28, 0.38]	0.30^**^ [0.25, 0.35]	0.35^**^ [0.30, 0.40]	
8. Voting intention	84.41	29.63	−1.87	2.13	0.12^**^ [0.06, 0.17]	0.02 [−0.03, 0.08]	0.01 [−0.05, 0.06]	0.05 [−0.01, 0.10]	−0.00 [−0.06, 0.05]	0.30^**^ [0.25, 0.35]	0.28^**^ [0.23, 0.33]

Values in square brackets indicate the 95% confidence interval for each correlation; ^*^p < 0.05, ^**^p < 0.01, ^***^p < 0.001. Means, standard deviations, skewness, kurtosis, and correlations with confidence intervals.

### 5.1 Social media news use and subjective knowledge

Our results show that the level of motivation in consuming news on social media is key to how people engage in reasoning: Stronger motivation, as measured by in intentional news search, is more strongly related to both political talk and intrapersonal reasoning (as measured by subjective knowledge) than incidental exposure. These findings support the premise of the peripheral elaboration model (Shahin et al., 2021) that motivation is critical for the following level of elaboration. This model proposes that intentional exposure triggers the central processing route leading to more elaboration, whereas incidental exposure triggers the peripheral route leading to less elaboration (Petty and Cacioppo, 1986). Both pathways can be observed in our findings.

The connection between intentional news search, political talk, and subjective knowledge can be explained by the concept of self-effects, in the sense that people’s behavior informs their self-concepts. People observe their behavior and infer information about themselves from it (Valkenburg, 2017; Ward et al., 2022). Thus, talking about politics could lead to the inference that one is knowledgeable about politics. The very act of discussing politics seems to serve as a heuristic for self-assessment. Even if it does not increase objective knowledge, it could still create the impression of knowledge gains, which could even extend beyond the topics discussed. This is especially true for public action (Ward et al., 2022). If people remember that they often searched for news on social media, they might interpret this as a sign that they are becoming more politically knowledgeable, but it lacks the public accountability that political talk has. Therefore, the self-effect through search may be lower than through political talk.

This relationship may also be explained by a different mediation sequence: It seems likely that those who feel more knowledgeable also feel more encouraged and comfortable to engage in political talk. Schäfer (2020) showed that subjective knowledge is related to a greater willingness to discuss and an increase in attitude strength, two important factors in political talk. Whether subjective knowledge fosters political talk or vice versa, or whether there is a reciprocal reinforcement operating is a key question for future research.

### 5.2 Social media news use and objective political knowledge

In contrast to previous research (Amsalem and Zoizner, 2022), as well as the O-S-R-O-R model (Cho et al., 2009), our findings suggest that general social media news use is negatively associated with objective knowledge. However, when we look more closely at the motivation for news consumption, we find that neither intentional news seeking nor incidental news exposure is directly related to objective knowledge. While the latter adds to the body on literature finding very mixed results on the relationship between incidental news exposure and objective knowledge (Oeldorf-Hirsch, 2018; Shehata and Strömbäck, 2021; Nanz and Matthes, 2022a), it contradicts a recent meta-study, showing a positive relationship (Nanz and Matthes, 2022a).

Even when political talk is included as a mediator, we find no positive relationship between intentional social media news use and objective knowledge. On the contrary, political talk is negatively related to objective knowledge, contrary to the predictions of the O-S-R-O-R model (Cho et al., 2009). It seems that while intentional social media use may lead to interpersonal reasoning in the form of political talk, the content that people talk about may not be rich enough to contribute to more knowledge. Political posts on social media often present political information as so-called “snack news” which contain little information and do not contribute to more objective knowledge (Schäfer et al., 2017; Schäfer, 2020). Another reason for the lack of objective learning may be that political talk does not occur in the same way in all social situations. Many studies that support an effect of political discussion on learning are set either in the offline world (Amsalem and Nir, 2019) or in online platforms, whose circumstances are significantly different from contemporary social media environments (Shah et al., 2017). In contrast to dyadic discussions, political expression on social media requires only low to moderate elaboration (Vaccari et al., 2015). However, since elaboration is key to learning (Eveland, 2001), the learning effects of social media use are mostly attributed to political talk with high elaboration or intentional news search (Hopp et al., 2023). Moreover, political talk is not only driven by political motivations, but also by people’s desire to maintain or strengthen relationships with others (Gil de Zúñiga et al., 2014, 2016; Neubaum, 2021). This could lead to less elaborate or thoughtful political talk.

However, this cannot explain why we found a negative association between political talk and objective political knowledge, both within Model 2 and as a bivariate correlation. There are several possible explanations. First, a certain level of like-mindedness and opinion congruence in one’s social media environment might - in the long run - hinder one’s knowledge acquisition (Cacciatore et al., 2018). Engaging in discussions in those politically homogeneous spaces could reduce people’s objective knowledge (Van Aelst et al., 2022). In addition, while news seeking shows openness to new information, news sharing is associated with reduced receptivity to new topics, possibly preventing learning (Martin and Sharma, 2023). This could indicate a displacement effect of news sharers preferring social media as a news platform over traditional news platforms. However, the latter, is better suited to promote learning effects (Lee et al., 2022). Second, relying on the frequency of political talk rather than the content of those discussions could lead to a mismatch between the topics of discussion and the topics we measured to operationalize objective knowledge. Because people mostly discuss hot-button issues (Maier, 2010), this could reduce the ability to learn about more complex issues.

The negative relationship between social media news use and objective knowledge could be explained by two factors. First, the validity of political knowledge as a construct, compared to other knowledge domains, is highly dependent on the items used to measure it (Burnett, 2016). It is possible that social media informs people about certain issues, while leaving them unaware of most other political news. Traditional media do not present information in isolation (e.g., the name of a political candidate), but include a variety of background information (e.g., the candidate’s party, their political ideology, and policy proposals). In addition, people may absorb news on related topics, which is why high-quality traditional media are associated with high levels of objective knowledge (Lee et al., 2022). In social media, however, information can be presented in isolation and is subjective to the curation of filtering algorithms. Second, it could indicate a displacement effect. People who feel well informed by social media may consume less traditional media, which could provide them with more objective knowledge (Lee et al., 2022). Nevertheless, we should not overestimate this relationship as it explains only 1% of the variance in objective knowledge. Any additional variance in Model 2 is due to the introduction of control variables (see Supplementary Figure S1). Therefore, we interpret our results as largely consistent with the lack of objective knowledge gain from social media shown by Amsalem and Zoizner (2022).

Our detailed examination of the role of motivation in mediating knowledge effects showed that not all forms of social media news use are equal and have equal connections to objective and subjective political knowledge. Furthermore, it adds to the argument that looking at broad social media news may be less informative, if not misleading, as it suggests that social media news use is negatively related to objective knowledge. Based on our findings, only motivated social media use is more strongly related to both objective and subjective knowledge. The question arises: What must happen during these motivated forms of use to support people’s political knowledge acquisition?

### 5.3 The mismatch between objective and subjective knowledge

In addition to these main findings, this study is the first to contrast different dimensions of knowledge, namely surveillance and static knowledge, and their relationships with social media news use (see Supplementary Table S2). Looking at objective knowledge, we showed that all specific forms of use were negatively correlated with surveillance knowledge, while no relationship was found with static knowledge. This underscores the power of social media in shaping surveillance knowledge. However, in contrast to traditional media (Barabas et al., 2014), the relationship we found between social media and surveillance knowledge was negative (Amsalem and Zoizner, 2022). This could be due to insufficient coverage of surveillance news on social media, or that people with less surveillance knowledge choose social media as a news platform. Static knowledge, on the other hand, remains unrelated to social media use, as it can be commonly generated through formal education (Barabas et al., 2014). Our data contrasts the findings of a meta-study that found no moderating effect for any type of knowledge (Amsalem and Zoizner, 2022). Turning to subjective knowledge, all forms of getting news from social media are positively associated with subjective knowledge. Replicating the above findings, motivated news use, that is intentional news searching, shows a higher association with all forms of knowledge compared to general and incidental news use.

This study confirms previous findings, showing that social media news use is positively associated subjective knowledge but has a negative effect on objective knowledge. Several studies discuss this as a possible illusion of knowledge, as subjective knowledge increases faster than corresponding objective knowledge (Schäfer, 2020; Granderath et al., 2021; Lee et al., 2022). This may be a challenge for democracy, as subjective knowledge is associated with polarization indicators such as anti-establishment voting, willingness to discuss and attitude strength (Schäfer, 2020; van Prooijen and Krouwel, 2020).

### 5.4 Effects on voting intentions

Previous findings have suggested that social media news use can increase political participation (Boulianne, 2020; Lee et al., 2022). While we expected similar results for voting intentions, no form of social media news use showed a direct relationship with voting intentions. There are two possible explanations. First, the O-S-R-O-R model, among others, describes media effects as mediated by reasoning (Eveland, 2001; Sotirovic and McLeod, 2001; Cho et al., 2009). By adding subjective knowledge as an emergent outcome of intrapersonal reasoning, this study shows that it can mediate the relationship between social media and voting intentions. Although knowledge is important for voting (Smets and van Ham, 2013), people may not always accurately assess their knowledge. Subjective knowledge can serve as a proxy, indicating that one is knowledgeable enough to vote based on their knowledge (Almond and Verba, 1965; Kaid et al., 2007; Dunning, 2011). Regardless of how people use social media to receive news, it is associated with an increase in subjective knowledge, and thus could promote voting intentions. Second, many forms of political participation require resources (Verba and Nie, 1972). Social media can reduce barriers to political participation through the affordances of social media. Online political participation, such as commenting and liking, requires fewer resources than joining a political party or attending a demonstration (van Deth, 2016; Knoll et al., 2020). Online political participation has increased with the advent of social media technologies (Boulianne, 2020). However, in most countries, voting is an offline, high-effort activity. Therefore, we expected social media news use to promote voting through mobilization, such as reminding people to vote, establishing voting norms (Arceneaux and Nickerson, 2009), or providing knowledge (Verba and Nie, 1972; Smets and van Ham, 2013). Still, our research suggests that social media cannot sufficiently reduce barriers, at least not directly.

Objective knowledge is important for one’s voting intention. This confirms previous evidence that objective knowledge is an important predictor of voting intention (Smets and van Ham, 2013; van Deth, 2016). Furthermore, it shows that, in particular, one’s static general knowledge (on both the objective and subjective dimensions) is predictive of voting intentions (see Supplementary material). Since knowledge about the basic foundations of democracy and the electoral system is classified as static general knowledge, this finding is not surprising, but important in light of declining voter turnout.

### 5.5 Limitations and outlook

Our research has several shortcomings. Although we intended to cover different dimensions of objective and subjective knowledge, we cannot rule out the possibility that other relevant dimensions play an important role in these processes. Obviously, no survey can capture objective political knowledge as a whole or all the specific issues that are important to individuals. We also did not assess campaign knowledge, but we assume that this form of knowledge tends to influence one’s specific vote choice, not voter turnout. While this study advances the way objective knowledge is operationalized in most studies, it cannot uncover hidden biases due to political identity or guessing. Follow-up studies could apply signal-detection-theory to uncover these (Nelson et al., 2013; Beattie and Milojevich, 2023). Unlike objective and subjective knowledge, political talk, which is involved in reasoning, was measured only at a very broad level. In addition, we measured voting intentions that were higher than the final voter turnout in NRW (Bundeswahlleiter, 2022).

Cross-sectional studies cannot account for causal relationships and the correlations found can be interpreted in different directions. For example, social media news use may not only promote political participation, but the two might also be interrelated (Lee and Xenos, 2022). In addition, subjective knowledge might not only influence people’s voting intentions, but forming voting intentions might lead people to perceive themselves as more knowledgeable (Schäfer, 2020; Ward et al., 2022). However, as the election took place after the study ended, it is more plausible that social media news use and subjective knowledge would impact voting intentions than the other way around.

Incidental news exposure and intentional news search share some variance. This is not surprising, as both are news-oriented behaviors on the same platform. Nevertheless, it may be difficult for participants to clearly distinguish between different forms of news use on social media (Oeldorf-Hirsch, 2018). As mentioned above, we asked participants about their incidental exposure, not their processing of these news. This might explain why we did not find an association with either form of knowledge. We decided to measure social media use using previous operationalizations. However, this measure is very broad largely neglecting whether there is an intention to expose to news. Given the breadth of this variable, results need to be interpreted with caution.

To better understand how social media news use leads to subjective knowledge and to establish causality, future studies should experimentally manipulate different forms of social media news use. In particular, eye-tracking studies could help to understand incidental news exposure and how it might generate subjective knowledge (Nanz and Matthes, 2020). To further disentangle the possible illusion of knowledge, future studies could focus on better aligning objective and subjective knowledge. To this end, the present work offers a solution by measuring both forms of knowledge along temporal and topical axes. In addition, Hopp et al. (2023) recommend distinguishing between political expression (liking and sharing of posts) and political talk (commenting and discussing), as this would help to distinguish between high and low effort elaboration.

## 6 Conclusion

This study confirms existing findings showing that the relationship between social media news use and political participation is mediated through different forms of processing this political news. Extending the state of knowledge, our findings emphasize that it is crucial to consider the level of intention with which users consume political news, as different levels of intention entail different levels of cognitive reasoning. Extending the O-S-R-O-R model (Cho et al., 2009) and its focus on the “orientation” people bring to the table when consuming news, we provide different manifestations of *motivational* orientation by distinguising between intentional and incidental news exposure and show that both are differently associated with political knowledge. Furthermore, we highlight the importance of “reasoning” for explaining the effect of media on political participation. Only when social media news consumption is associated with political talk or an increase in people’s subjective knowledge can it increase people’s voting intentions.

This study informs lines of inquiry that seek to uncover the circumstances under which news-related social media use can have real-world impacts and actual effects on democratic processes. Social media seem to be less important for informing people than for facilitating political discussions and strengthening their (political) self-concepts and subjective knowledge, which in the long run could increase the likelihood of participating in political processes by voting. However, these positive effects seem to depend on people’s motivation to seek out and engage with this news in-depth.

## Data availability statement

The datasets presented in this study can be found in online repositories. The names of the repository/repositories and accession number(s) can be found in the article/Supplementary material.

## Ethics statement

The studies involving humans were approved by Ethikkommission der Abteilung Informatik und Angewandte Kognitionswissenschaft der Fakultät für Ingenieurwissenschaften der Universität Duisburg-Essen. The studies were conducted in accordance with the local legislation and institutional requirements. The participants provided their written informed consent to participate in this study.

## Author contributions

JD and GN contributed to the conception and the design of the study. JD performed the statistical analyses and wrote the first draft of the manuscript. All authors contributed to the article and approved the submitted version.

## Funding

We acknowledge support by the Open Access Publication Fund of the University of Duisburg-Essen.

## Footnotes

^1^Compared to the pre-registration, we have changed the wording of some hypotheses and research questions, but not their meaning (see Supplementary material).

^2^In addition, we asked research questions about different categories of objective and subjective knowledge (see Supplementary material for wording and analyses).

^3^ https://osf.io/s5aq9

^4^Due to poor model fit, we excluded all questions that asked about news use on specific platforms. See Supplementary material for frequency of platform use.

^5^Not all participants who said they had already voted by mail stated 100% certainty to vote. Recoding the responses of these participants to a 100% certainty resulted in little to no changes in the following analyses. Similar results were obtained when also excluding all participats who had already voted.

^6^See Supplementary Figure S1 for Model 2 without control variables.

^7^We applied Hittner et al.’s (2003) modification of Dunn and Clark’s (1969) using a back transformed average Fisher’s (1921) Z procedure in R.
