# Corrigendum: How incidental and intentional news exposure in social media relate to political knowledge and voting intentions

**DOI:** 10.3389/fpsyg.2024.1476279

**Published:** 2024-08-30

**Authors:** Jana H. Dreston, German Neubaum

**Affiliations:** Department of Human-Centered Computing and Cognitive Science, University of Duisburg-Essen, Duisburg, Germany

**Keywords:** subjective knowledge, political knowledge, social media, election, incidental news exposure

In the published article, there was an error in Figure 1. The figure consists of four boxes, whereby the middle boxes were incorrectly both labeled “Objective Knowledge”. The bottom middle box should be labeled “Subjective Knowledge” instead. The corrected Figure 1 and its caption appear below.

**Figure 1:**
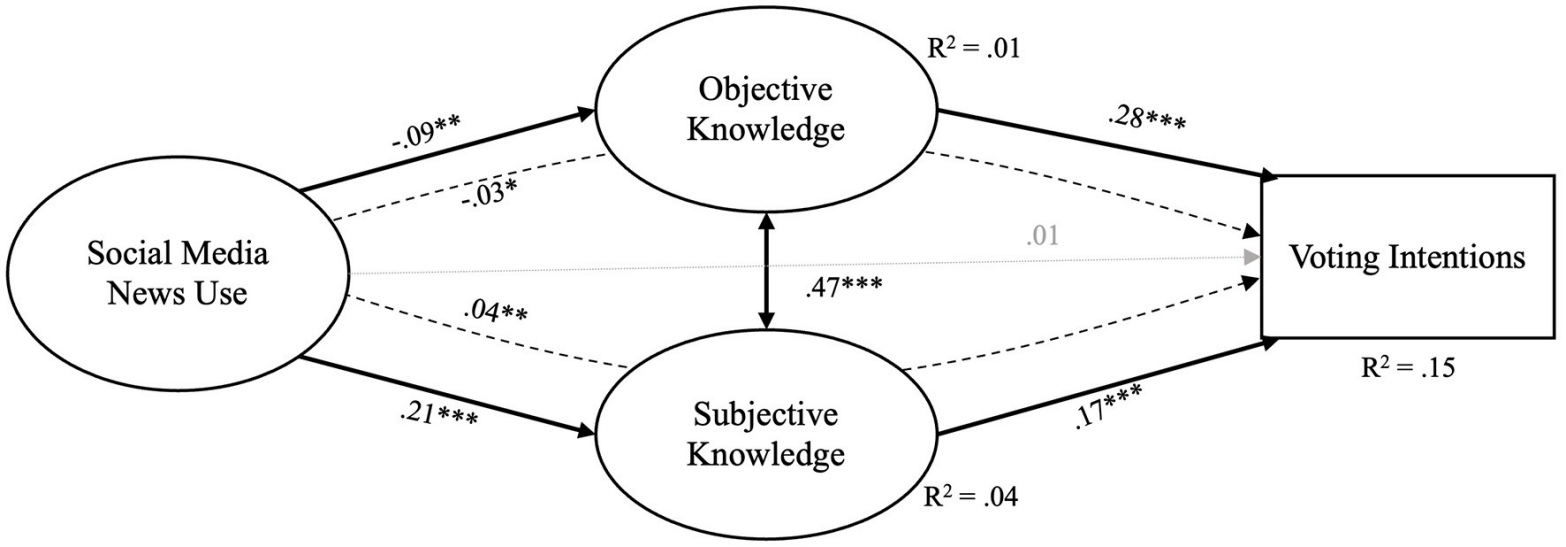
Model 1. Model fit: χ^2^(270) = 366.55, *p* < 0.001, χ^2^/df = 1.75, CFI = 0.98, RMSEA = 0.03 (90% CI: 0.02, 0.03), SRMR = 0.03; *p* < 0.05 (^*^), *p* < 0.01 (^**^), *p* < 0.001 (^***^); Full lines indicate significant direct associations, dashed lines indicate indirect significant associations, dotted lines indicate non-significant association.

The authors apologize for this error and state that this does not change the scientific conclusions of the article in any way. The original article has been updated.

