# Supplementary material for: How incidental and intentional news exposure in social media relate to political knowledge and voting intentions
Source: Front Psychol. 2023 Dec 21;14:1250051. doi: 10.3389/fpsyg.2023.1250051 (PMC10768061; doi:10.3389/fpsyg.2023.1250051)
Supplement: Supplementary file 1 [file Data_Sheet_1.pdf]

Supplementary Material

How Incidental and Intentional News Exposure in Social Media Relate to Political Knowledge and Voting Intentions

Jana H. Dreston*, German Neubaum

*** Correspondence:** Jana Dreston: jana.dreston@uni-due.de

All data used in this study can be accessed via: https://osf.io/yhe6n/

1 Supplementary Figures

1.1 **Figure 1.** Model 2 without control variables.

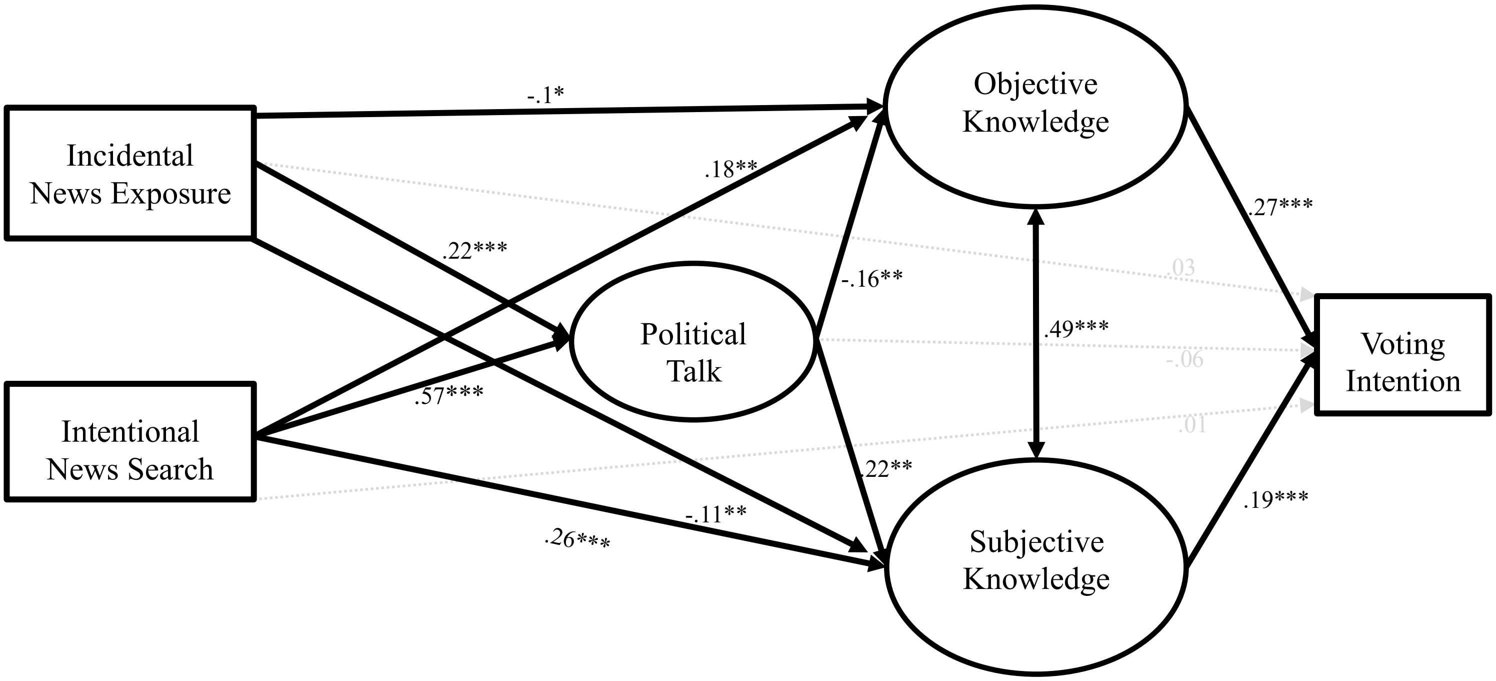

Note. Model Fit: $\chi^2(218) = 517.38$, $p < .001$, $\chi^2/df = 2.37$, CFI = .97, RMSEA = .03 (90% CI.: .03, .037), SRMR = .03;); p < 0.05 (*), p < 0.01 (**), p < 0.001 (***);full lines indicate significant direct associations.

1.2 Figure 2. Incidental News Exposure Model.

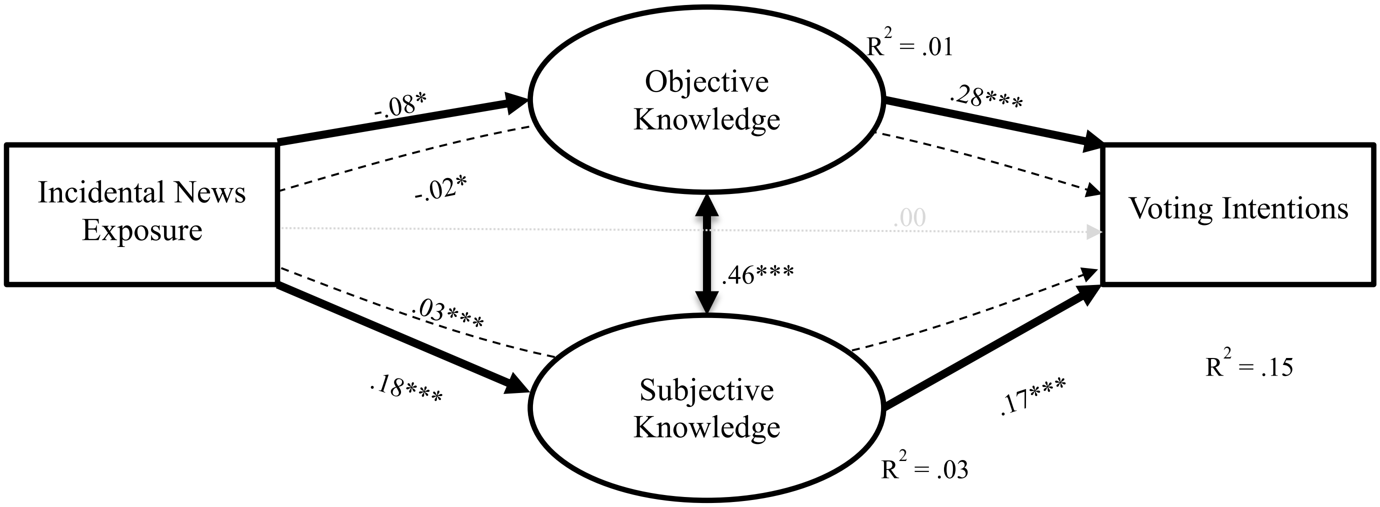

Note. $\chi^2(148) = 345.73$, $p < .001$, $\chi^2/df = 2.34$, CFI = .97, RMSEA = .03 (90% CI.: .03, .04), SRMR = .03; full lines indicate significant direct associations, dashed lines indicate indirect significant associations, dotted lines indicate non-significant association.

1.3 **Figure 2.** Intentional News Search Model.

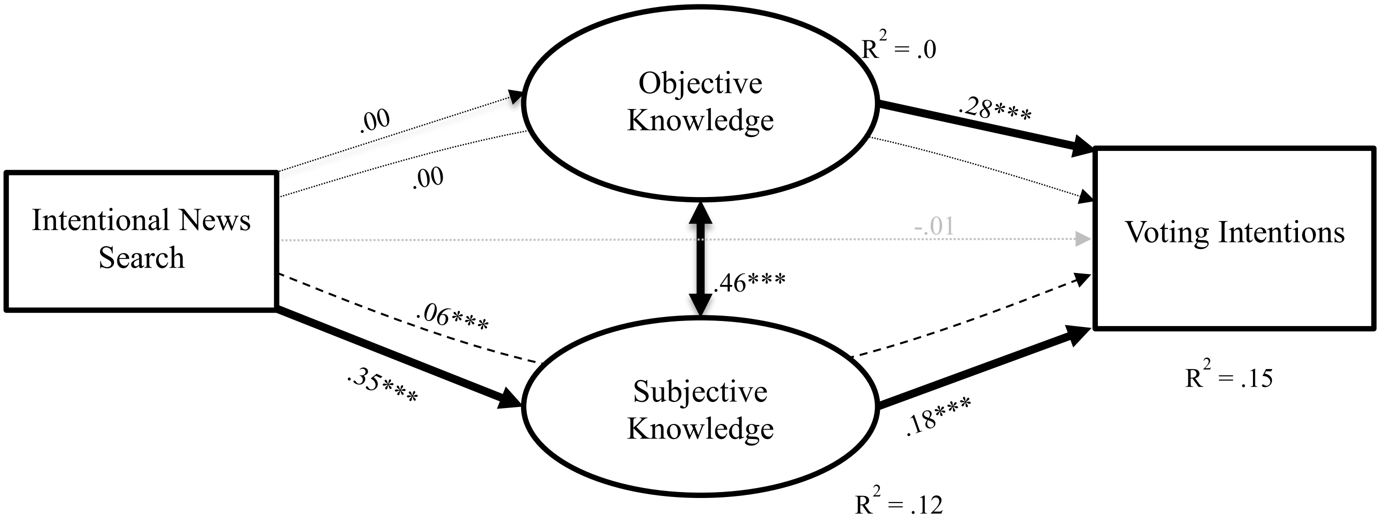

Note. $\chi2(148) = 338.12$, $p < .001$, $\chi2/df = 2.28$, CFI = .97, RMSEA = .03 (90% CI.: .03, .04), SRMR = .03; full lines indicate significant direct associations, dashed lines indicate indirect significant associations, dotted lines indicate non-significant association.

1.4 **Figure 4.** Political Talk Model.

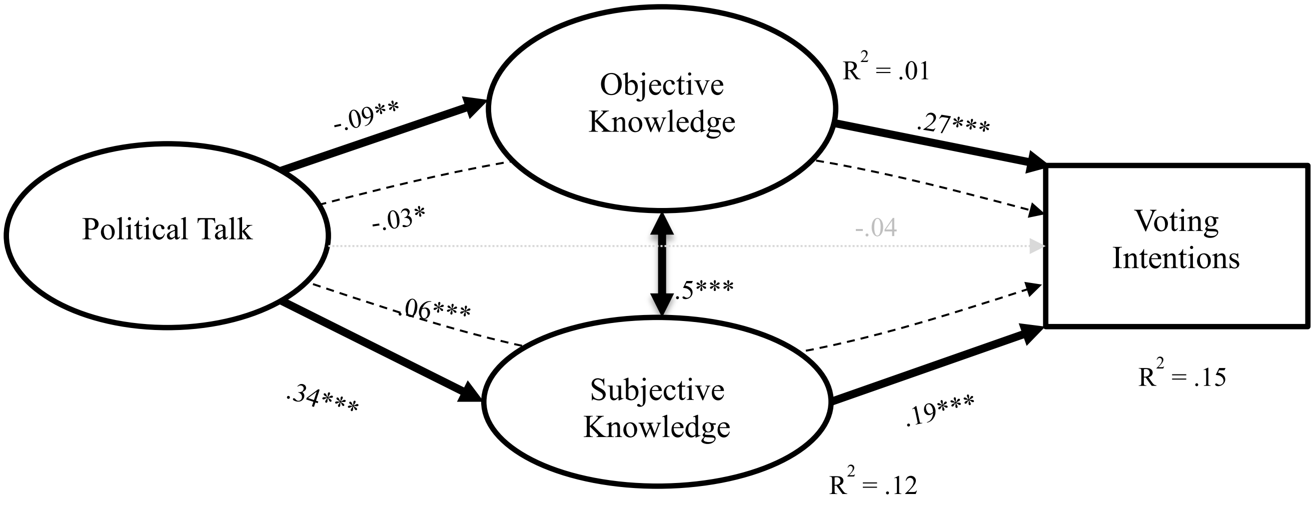

Note. Model Fit: $\chi^2(184) = 397.37$, $p < .001$, $\chi^2/df = 2.16$, CFI = .98, RMSEA = .03 (90% CI.: .03, .04), SRMR = .03); p < 0.05 (*), p < 0.01 (**), p < 0.001 (***); full lines indicate significant direct associations, dashed lines indicate indirect significant associations, dotted lines indicate non-significant association.

2 Supplementary Tables

2.1 **Table 1.** Correlation between sub-categories of objective and subjective knowledge.

			**Subjective Knowledge**			
			Static		Surveillance	
			General	Policy	General	Policy
**Objective Knowledge**	Static	General	.38***	.28***	.33***	.36***
		Policy	.17***	.15***	.14***	.17***
	Surveillance	General	.29***	.19***	.26***	.26***
		Policy	.22***	.13***	.19***	.19***

Note. †p < .10, ∗p < .05, ∗∗p < .01, ∗∗∗p < .001.

2.2 Table 2. Correlation between forms of social media news use and sub-categories of objective and subjective knowledge

			Social Media News Use	Incidental News Exposure	Intentional News Search	Political Talk
Objective Knowledge	Static	General	-.04	-.05	.0	-.06
		Policy	-.06*	-.01	.0	-.04
	Surveillance	General	-.07*	-.09**	-.01	-.1***
		Policy	-.10***	-.09**	-.06*	-.11***
Subjective Knowledge	Static	General	.15***	.15***	.3***	.29***
		Policy	.15***	.17***	.25***	.21***
	Surveillance	General	.18***	.16***	.27***	.28***
		Policy	.18***	.17***	.32***	.27***

Note. †p < .10, ∗p < .05, ∗∗p < .01, ∗∗∗p < .001.

2.3 Table 3. Correlation between sub-categories of objective and subjective knowledge and voting intentions.

	Static		Surveillance	
	General	Policy	General	Policy
Objective Knowledge	.27***	.18***	.24***	.21***
Subjective Knowledge	.28***	.2***	.24***	.27***

Note. †p < .10, ∗p < .05, ∗∗p < .01, ∗∗∗p < .001.

2.4 Table 4. Social Media Platforms.

Platform	Mean	Standard deviation
Facebook	3.67	2.08
Twitter	2.18	1.7
Instagram	3.74	2.21
TikTok	2.24	1.91
Youtube	4.04	1.81

Note. Use of social media platforms for news

2.5 **Table 5.** List of all pre-tested knowledge questions.

All items used in study are in bold. We excluded some items due to problems in the factor analysis (italic).

Temporal axis	Topic axis	Regional focus	Original question	Translated question	Correct answers In %
Static	General	Germany	***Welche Funktion hat die Zweitstimme bei der Bundestagswahl?***	***What is the function of the second vote in the federal German election?***	**0.69**
			Was besagt die Sperrklausel für Sitze in Landes- und Bundestagen?	What is the threshold for seats in the state and federal parliament?	0.9
			Wie heißt der direkte Vorgänger Annalena Baerbocks als Außenminister?	What is the name of Annalena Baerbock's direct predecessor as Foreign Minister?	0.69
			Wie heißt der aktuelle Bundespräsident?	What is the name of the current German President?	0.81
			**Welche ist die älteste Partei im deutschen Bundestag?**	**Which is the oldest party (by year of foundation) in the German parliament?**	**0.69**
			Welche Aussagen zu der Ministerpräsidentin Giffey stimmt?	Which statement about Minister President Giffey is true?	0.78
		NRW	**Welche Parteien bilden gerade die Regierungskoalition in NRW?**	**Which parties are currently forming the governing coalition in NRW?**	0.6

			In welcher Stadt fand das Loveparade Unglück 2010 statt?	In which city did the Love Parade disaster take place in 2010?	0.83
			Der Landtag von NRW befindet sich in welcher Stadt?	The state parliament of NRW is in which city?	0.88
			**Aus welcher Stadt stammt der erste Bundeskanzler Konrad Adenauer?**	**From which city was the first German chancellor Konrad Adenauer?**	**0.55**
			Die letzte SPD geführte Regierung in NRW gab es unter?	The last government in NRW with an SPD majority was led by whom?	0.71
Static	Policy	Germany	***Seit wann dürfen homosexuelle Paare in Deutschland heiraten?***	***In which year did same-sex-marriage become legal in which year in Germany?***	**0.52**
			Wie hoch ist der Regelsatz der Mehrwertsteuer in Deutschland?	What is the standard rate of VAT in Germany?	0.9
			Welche Aussage zur doppelten Staatsbürgerschaft stimmt? Eine doppelte Staatsbürgerschaft…	Which statement about dual citizenship is true? A dual citizenship...	0.59
			**In welchem Jahr kündigte Angela Merkel die Abkehr von der Atomkraft an?**	**In which year did Angela Merkel announce the ending of nuclear power stations in Germany?**	**0.62**
			In welchem Jahr fand die Wiedervereinigung statt?	In which year did the German reunification take place?	0.74
			Wie hoch ist das Rentenalter für Menschen, die nach 1964 geboren sind?	What is the retirement age for people born after 1964?	0.83

		NRW	**Aus welchem Grund wurde die Räumung von Demonstrationen im Hambacher Forst 2018 geräumt?**	**For what reason was the evacuation of demonstrations in Hambacher Forst ordered in 2018?**	**0.5**
			Die Umstellung von G8 auf G9 in Gymnasien in NRW…	***The conversion from G8 to G9 in grammar schools in NRW...***	**0.62**
			Im September 2021 wurde in Hagen ein Anschlag vereitelt. Worauf war dieser Anschlag geplant?	In September 2021, an attack was impeded in Hagen. What was the purpose of this attack?	0.72
Surveillance	General	Germany	Welcher Ministerpräsident wird im Mai 2022 vorzeitig sein Amt niederlegen?	Which state-minister will resign early in May 2022?	0.52
			**Welche deutsche Politikerin / welcher deutsche Politiker infizierte sich im Ausland mit Corona?**	**Which German politician got infected with Corona abroad?**	**0.5**
			Wie heißt der oder die im März im Saarland abgewählte Ministerpräsident/die Ministerpräsidentin?	What is the name of the state-minister who was voted out of office in Saarland in March?	0.53
			Welche Politikerin/welcher Politiker verließ vor kurzem seine Partei?	Which politician recently left their party?	0.52
			Welche Politikerin steht wegen North Stream 2 gerade in der Kritik?	Which politician is currently being criticised because of North Stream 2?	0.67
			Wie heißt die neue Familienministerin?	What is the name of the new family minister?	0.59

			Wie heißt der neue Botschafter in Israel?	What is the name of the new ambassador to Israel?	0.71
			Was macht die Abgeordnete Tessa Ganserer so besonders?	What makes MP Tessa Ganserer so special?	0.84
			Von welchem Ministerinnen Posten trat Anne Spiegel zurück?	From which ministerial post did Anne Spiegel resign?	0.84
			Anne Spiegel trat auf Grund von Fehltreten während ihrer Zeit als Ministerin in Rheinland-Pfalz zurück. Welchen Ministerinnenposten hatte sie damals inne?	Anne Spiegel resigned due to absenteeism during her time as minister in Rhineland-Palatinate. Which ministerial post did she hold at the time?	0.55
		NRW	Welche Politikerin/welcher Politiker war für Wirtschaftsverhandlungen kürzlich in Katar?	Which politician recently visited Qatar for economic negotiations?	0.84
			**Wie heißt der Spitzenkandidat der SPD für die Landtagswahl?**	**What is the name of the SPD's front-runner for the state election?**	**0.69**
			**Der größte Waffenhersteller Deutschlands kommt aus NRW. Wie heißt dieser?**	**The largest arms manufacturer in Germany produces in NRW. What is its name?**	**0.55**
			Welche in NRW ansässige Firma steht wegen Ihrer Handlungen im Zusammenhang mit dem Ukraine Krieg in der Kritik?	Which NRW-based company has been criticised for its actions in connection with the Ukraine war?	0.26
			Was soll in Zukunft erlaubt sein?	What is going tob e allowed in the future?	0.9

Surveillance	Policy	Germany	Wie teuer soll das vergünstigte ÖPNV-Ticket, als Reaktion auf die gestiegenen Ölpreise, werden?	How expensive should the discounted public transport ticket become (in response to the rise in oil prices)?	0.91
			**Wo wurde das erste Tesla Werk Europas vor kurzem eingeweiht?**	**Where was Europe's first Tesla factory recently open?**	**0.62**
			Über welchen Energieträger hat der deutsche Wirtschaftsminister in Katar verhandelt?	Which energy source did the German Minister of Economics negotiate in Qatar?	0.28
			**Wie hoch ist das geplante Sondervermögen für die Bundeswehr?**	**How high is the planned special fund for the Bundeswehr?**	**0.81**
			Welche Coronamaßnahme wurde nach weniger als 24 Stunden von Minister Lauterbach wieder gekippt?	Which Corona action was overturned by Minister Lauterbach after less than 24 hours?	0.83
		NRW	**Welches Verkehrspolitische Projekt führt gerade zu großflächigem Verkehrschaos in der Region?**	**Which transport project is currently causing widespread traffic chaos in the region?**	**0.64**
			Das Oberverwaltungsgericht Münster urteilte kürzlich...	***The Münster Higher Administrative Court recently ruled that...***	**0.48**
			Vor kurzem wurde eine Rückholaktion für Frauen, unter anderem aus NRW gestartet. Welcher Terrororganisation hatten sie sich angeschlossen?	Recently, a repatriation campaign was launched for women, among others from NRW. Which terrorist organisation had they joined?	0.78

Table 6. Point estimates and 95% confidence intervals for all indirect effects

			95% CI	
Indirect Effect Path	Point Estimate	SE	LL	UL
Model 1				
Social Media News Use → Objective Knowledge → Voting Intentions	-.03 (*p*=.011)	.01	-.05	-.01
Social Media News Use → Subjective Knowledge → Voting Intentions	.04 (p<.001)	.01	.02	.06
Model 2				
Political Talk → Objective Knowledge → Voting Intentions	-.06 (*p*<.001)	.02	-.1	-.03
Political Talk → Subjective Knowledge → Voting Intentions	.02 (*p*=.003)	.01	.01	.04
Intentional News Search → Political Talk → Objective Knowledge → Voting Intentions	-.03 (*p*=.001)	.01	-.05	-.02
Intentional News Search → Political Talk → Subjective Knowledge → Voting Intentions	.01 (*p*=.01)	.005	.00	.02
Incidental News Exposure → Political Talk → Objective Knowledge → Voting Intentions	-0.01 (*p*=.001)	.07	-02	-.01
Incidental News Exposure → Political Talk → Subjective Knowledge → Voting Intentions	.01 (*p*=.01)	.00	.00	.001

Note. Bootstrap N = 5000: Model 1 without control variables, Model 2 controlled for age, gender, education, and interest

3 Supplementary Documents

3.1 Documentation of altered hypotheses and research questions

We altered the wording of some hypotheses and research questions after pre-registration, without changing the meaning of the very.

List of new hypotheses

H1: The frequency of social media news use is positively associated with subjective political knowledge.

H2: Political talk on social media is positively associated with a) objective political knowledge and b) subjective political knowledge.

RQ1: Are incidental news exposure and intentional news search on social media differently associated with subjective political knowledge?

H3: Both a) social media news use and b) political talk are positively correlated with voting intentions.

RQ2: Are incidental news exposure and intentional news search differently associated with voting intentions?

H4: Subjective political knowledge is positively associated with voting intentions.

H5: Subjective political knowledge mediates the positive relationship between a) social media news use and b) political talk and voting intentions.

RQ3: Does subjective political knowledge mediates the positive relationship between a) incidental news exposure and b) intentional news search and voter turnout?

List of pre-registered hypotheses and research questions

H1: The frequency of social media news use is positively associated with subjective political knowledge

RQ1: Is the frequency of incidental and active news exposure on social media differently associated with subjective political knowledge?

H2: The frequency of news-related political talk on social media is positively associated with objective political knowledge.

H3: The frequency of news-related political talk on social media is positively associated with subjective political knowledge.

H4: Frequency of social media news use is positively correlated with likelihood of voting.

RQ2: Are the frequencies of incidental and active news exposure differently associated with the likelihood of voting?

H5: The frequency of news-related political talk on social media is positively associated with likelihood of voting.

RQ3: To what extent are the dimensions static general, static policy, surveillance general, surveillance policy of subjective political knowledge associated with different forms of social media use?

RQ4: To what extent are the dimensions static general, static policy, surveillance general, surveillance policy of objective political knowledge associated with different forms of social media use?

RQ5: To what extent are the dimensions static general, static policy, surveillance general, surveillance policy of objective political knowledge associated with the dimensions static general, static policy, surveillance general, surveillance policy of subjective political knowledge?

H6: Subjective political knowledge is positively correlated with likelihood of voting.

H7: Subjective political knowledge mediates the positive relationship between the frequency of social media news use and increased likelihood of voting.

RQ6: Does subjective political knowledge mediate the associations between incidental and active news exposure on likelihood of voting?

H8: Subjective political knowledge mediates the positive relationship between frequency of news-related political talk on social media and increased likelihood of voting.

RQ7: Does any dimension of objective knowledge (dimensions static general, static policy, surveillance general, surveillance policy) show any relationship with likelihood of voting?

Note. In the final paper, “active news exposure” is referred to as “intentional news search” and “likelihood of voting” is referred to as “voting intentions”.
